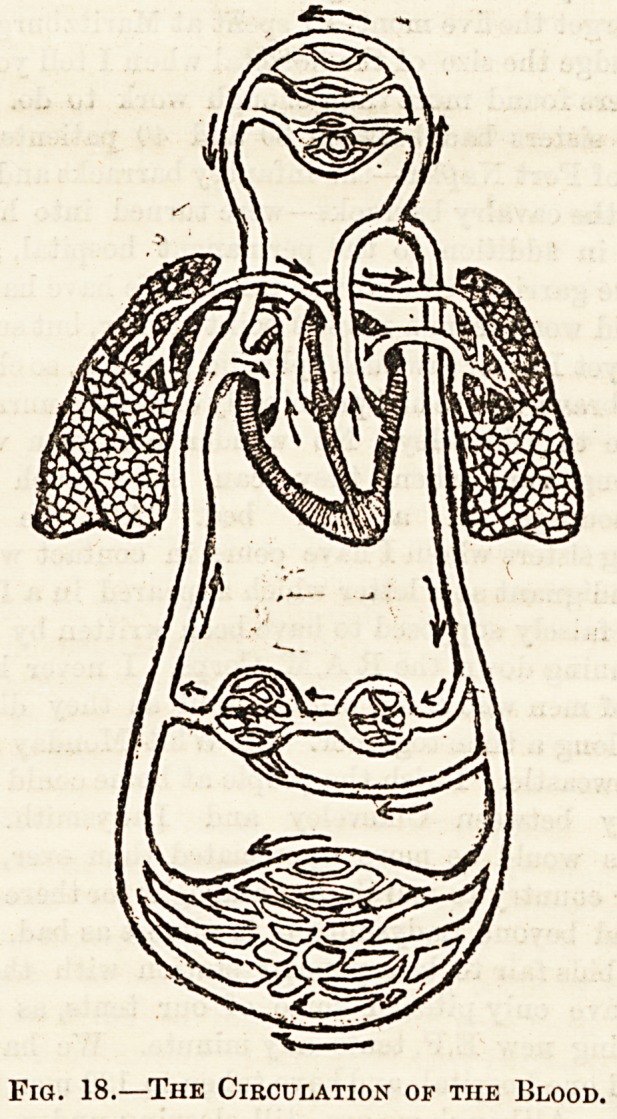# "The Hospital" Nursing Mirror

**Published:** 1900-07-07

**Authors:** 


"The Hospital" Nursing Mirror.

Being the Nursing Section of "The Hospital."

[Contributions for this Section of "The Hospital" should be addressed to the Editor, The Hospital, 28 & 29, Southampton Street, Strand, London, W.C., and should have the word "Nursing" plainly written in left-hand top corner of the envelope.]

### Notes on News from the Nursing World.

### THE ROYAL RED CROSS.

The *Gazette* announces that the decoration of the Royal Red Cross has been conferred by the Queen on Miss Mary Christina Anderson, nursing sister-in-charge of the Colonial Hospital at Suva, Fiji, for services rendered to British naval officers and men during the Samoa disturbances last year. We congratulate Miss Anderson upon the honour she has received at the hands of Her Majesty, which, as those who are familiar with her work are aware, is thoroughly well deserved.

### THE WAR OFFICE AND NURSING AT THE FRONT.

The serious indictment against the War Office by Mr. Burdett-Coutts, M.P., has promptly engaged the attention of the House of Commons, with the practical result that a Committee of Inquiry into the treatment of the sick and wounded in South Africa is to be immediately appointed. It is obviously of the utmost importance that at least one woman of experience in nursing should be a member of the Committee. With regard to the allegations of the member for Westminster, he has clearly proved that the number of nurses was, at the time of his visit to Bloemfontein, entirely inadequate. Mr. Wyndham did not indeed attempt to prove the reverse. In fact, when he came to the question of nursing, he said, "the figures which I will now give as to the army nurses will, I know, excite displeasure." The figures he gives are not very clear, but at all events they afford the most conclusive testimony possible of the miscalculation made at the outset, and this was actually all that the Under Secretary for War had to say on that branch of the subject. Yet, as Mr. Burdett-Coutts, in pressing home his indictment both in the House and in an interview with our Commissioner, insisted, the evils which occurred at Bloemfontein "were owing to the absence of a proper female nursing staff." We notice that it has since been urged in defence of the War Office that a sufficient number of fully trained female nurses could not have been obtained. This is absurd. The number of nurses willing and anxious to go to South Africa has from the first been very far in excess of the demand; and although, of course, a proportion of these would not be suitable for the work, it is really quite impossible to plead a dearth of recruits for the Army Nursing Reserve as an excuse for not sending out a supply large enough to deal with any emergency that might arise.

### THE NURSES OF THE IMPERIAL YEOMANRY BRANCH HOSPITAL.

The following members of the Army Nursing Service Reserve sailed in the "Norman" for South Africa on Saturday, to take up duty with the Imperial Yeomanry Branch Hospital: Miss Nisbet, East Dulwich Infirmary, matron; Miss A. M. Britten; Miss M. Brough, Royal Infirmary, Derby; Miss M. E. Hainselin, South Devon Hospital, Plymouth; Miss E. C. Rider, Lambeth Infirmary; Miss A. Rogers, St. George's Hospital; Miss E. K. Sharp, Royal Berks Hospital, Reading; Miss E. C. Smith; Miss F. Smith; Miss N. Templeton; Miss E. C. Thomas; and Miss L. Whiley, Royal Berks Hospital, Reading. The remaining 20 sisters sail on Saturday next. Miss K. E. Nisbet, the matron, was trained at King's College Hospital and the Children's Hospital, Great Ormond Street. She was matron of the hospital at Calcis, Greece, during the Græco-Turkish war, and has since been first assistant matron at St. Saviour's Infirmary, East Dulwich Grove. She holds the L.O.S. certificate. Miss Florence Smith was trained at St. Saviour's Infirmary, East Dulwich Grove, and nursed at Maidstone during the typhoid fever epidemic.

### THE WESTERN AUSTRALIAN NURSES' CONTINGENT.

The Australian papers contain letters from members of the Nurses' contingent for South Africa which indicate that they were treated with strange want of consideration by their own Government. For example, on the passage to the Cape several nurses were "accommodated" in temporary cabins in the hatchways, and all of them suffered various privations which should have been easily avoidable. Their diet appears to have consisted chiefly of biscuits, and on their arrival at Durban they were "thin and haggard for want of food." Then, again, when they got to Cape Town no one expected them, and they were obliged to wait for hours on the wharf, narrowly escaping the inconvenience of having to spend the night in the Customs House shed. At any rate, the nurses sent out of this country to the war have not had to complain of either the quantity or the quality of their food, or, indeed, of any of the arrangements made for their personal comfort. It is due to the other Colonies to add that Western Australia seems to be a unique example of "how not to do it."

### NURSING ON THE "MAINE."

In the report issued by Lady Randolph Churchill, as chairman of the American Hospital Ship Fund in South Africa, reference is made to the change in the nursing staff of the "Maine," which has just returned to England. Lady Randolph reiterates her anxiety that it should be fully understood that the only reasons why the services of Miss Hibbard and her colleagues were dispensed with were because the nursing staff was too large for the number of patients taken on board, because the bulk of the patients are men who are able to do a good deal for themselves, and because of the cramped accommodation and want of privacy the ship affords for women. On the point as to whether women or men are more valuable in the field, base hospitals, and hospital ships, Lady Randolph declines to express an opinion, but observes, "There is one thing certain, however, we could not do without the Sisters had we not equally skilled men nurses to replace them." Of the Sisters, Lady Randolph says "they did not shirk their duties, but carried them out with ability, giving to the wards that touch of grace and attractiveness which a woman alone can give."

### ST. BARTHOLOMEW'S HOSPITAL.

The nurses of St. Bartholomew's Hospital are deeply interested in the settlement of the site question which came before the meeting over which the Prince of Wales presided on Monday. One of the most pressing necessities is a new nursing home; for, as the treasurer, Sir Trevor Lawrence, said in his speech, "the nurses, a most valuable body of women, are at present scattered about, some here and some there, and there is no proper arrangement for their living in comfort." This is one of many reasons why it will be generally hoped that the Governors of St. Bartholomew's will obtain the land they need for extensions.

### A SWEEPING CHARGE AGAINST LONDON HOSPITALS.

According to a report in the *Christian World*, Dr. Horton, preaching on Hospital Sunday at Hampstead, said that "the matron of a hospital had lately told him that the training in any of the great London hospitals was so exacting and laborious that it added ten years to the age of those who passed through it, while many retired from it with permanently broken health." Dr. Horton went on to express the hope that none of the money contributed to the Hospital Fund would go to hospitals "which treated their nurses with such uncalled-for harshness." Undoubtedly, any hospital which treats its nurses harshly is unworthy of public support, but the charge which Dr. Horton puts forward on the authority of a matron is manifestly ridiculous, and will not, we trust, receive any credence. Those who are acquainted with the details of the training "in any of the great London hospitals" know perfectly well that while it is in many ways trying at first, later on it demands simply the exercise of intelligence, application, and love of the work, and is far from corresponding to the character described by Dr. Horton's matron.

### LORD DUFFERIN AND THE BANGOR NURSES.

The annual meeting of the Bangor District Nursing Society—which is affiliated with the Queen Victoria Jubilee Institute—was held last week under the presidency of the Marquis of Dufferin and Alva. The number of cases nursed during the year was 138, of which 91 had recovered, and, owing to the efforts made by the lady collectors, the society had a balance of £10 in hand. Lord Dufferin, in paying a high tribute of praise to Miss Martin, the district nurse, who was leaving Bangor, said that she would be followed by the "blessings of hundreds upon hundreds of poor people—men and women whom she had nursed through their desperate illnesses, and pains, and numerous trials." He graphically described the ministrations of a district nurse, and stated that "whatever home a nurse entered there entered with her a multitude of blessings—fresh air, sunshine, cleanliness, punctuality, order, and above all, consideration for others; in fact, most of the principal elements which constitute the happiness of a home."

### A STRANGE OFFER TO A NURSING HOME.

The Bilston District Nursing Association are not to derive advantage from the curious offer of Mr. James Dangerfield. This gentleman proposed to give his house to the association on condition that a local fund of £2,000 should be raised for the relief of the famine in India. He himself promised to contribute £100, but the terms of the proposed gift were not fulfilled, and the house was sold on Monday last. We conclude that Mr. Dangerfield imagined that members of the nursing association would set to work to obtain contributions for the Indian Famine Fund in order to secure possession of his residence, and so lessen the amount which would have to be subscribed to keep the association going in the future. They can hardly be blamed because they failed to see the advantage of an arrangement which would probably have resulted in benefiting the Famine Fund at the expense of the nursing association.

### NURSES FOR THE MIDDLE CLASSES.

Our excellent contemporary, the *Queen*, contains an interesting article on the need of nurses for the middle classes. The poor, it observes, are supplied with gratuitous nurses by charitable and parochial associations. The rich obtain their nurses at any cost; the middle classes are those who at the present time, unfortunately, have great difficulty in getting nursing assistance. The *Queen* goes on to account for this on the ground that middle-class families cannot afford to pay a nurse at the ordinary rate which is now charged by the nursing associations; and, in order to make provision for their requirements, it seems to suggest that persons of inferior training should be employed as nurses, and quotes the opinion of a medical journal, "that there are thousands of cases in which the help of a nurse of good common qualities for a portion of the day or night would be invaluable." But why should the middle classes alone suffer from the disadvantage of being tended by untrained nurses? We observe that in the course of a speech at Kidderminster the other day, Lord Cobham said he believed "the time was approaching when every town and district and village in the country will have the services of trained nurses, and when they will be regarded as necessary as post offices or policemen." This is by no means a Utopian vision, and it is one which we shall do our best to assist in realising. There is no reason why the sick poor only should benefit from the formation of district nursing associations. If the middle classes will only give the moderate amount of financial support which they can fairly afford to such organisations, they should be entitled to, and should not shrink from asking for, the assistance of a nurse whenever they require one.

### AMERICAN NURSES STUDY SPANISH.

The American nurses who are employed at Manila find their work comparatively light. Day nurses are on duty from half-past seven a.m. to twelve noon, and from two p.m. to half-past six p.m. In some of the wards it is so arranged that one nurse goes home to early luncheon, returning at a quarter-past twelve p.m. to relieve the others for the two hours. Night nurses report for duty at half-past six p.m., and leave the wards at seven a.m. Ambulances are detailed to convey them to and from the "home" at midday, as the heat at that time is quite intense. As to recreation, many of the hard-earned dollars of the nurses go, Miss Frances McCurdy, in a letter from the Philipines, says, for carromatta hire, there being a number of places of interest within easy driving distance. Another diversion, still more expensive, is shopping; while trips up the Pasig River in the hospital launch are in high favour. Many of the nurses very wisely spend some of their spare time in studying the Spanish language, and several of them, Miss McCurdy mentions, already speak it quite fluently. Others take up the guitar, or the native instrument, the biguera, which somewhat resembles the mandolin.

### THE HOURS AT PRIVATE ASYULMS.

A correspondent asks in another column for our opinion respecting the hours of work at a small private asylum to which she is attached. The night duty lasts for thirteen and a half hours, and the day duty from half-past six a.m. till half-past nine p.m. There is an evening off in the week from six to half-past nine, and every Sunday morning and evening alternately. These hours are unquestionably long; and in days when it is not always easy to secure the most suitable people as asylum nurses, the keeper of a private asylum should, as a matter of self-interest, show more considera tion for the members of the staff.

### NURSING AT THE FRONT.

An Army Nurse writes: "I have been living under canvas now since March 17th, and am getting so attached to my tent that I am sure I shall want to keep it when my turn comes to go home. I do not think I shall ever forget the five months I spent at Maritzburg. You may judge the size of the hospital when I tell you that 32 sisters found more than enough work to do. Most of the sisters had between 30 and 40 patients. The whole of Fort Napier—the infantry barracks and about six of the cavalry barracks—were turned into hospital wards in addition to the permanent hospital, and at last the garrison church was taken. We have had some splendid work from a nurse's point of view, but such sad work; yet I never saw such splendid patients, so cheerful and so brave. I have had principally officers to nurse until I came to Chieveley. No wonder our men won in the long run when they can show such pluck and courage in a sick bed. All the army nursing sisters whom I have come in contact with are very indignant at a letter which appeared in a London paper, falsely supposed to have been written by one of us, running down the R A.M. Corps. I never knew a body of men who could work as well as they did, and for so long a time together. On Whit Monday we left for Newcastle. I wish the people at home could see the country between Chieveley and Ladysmith. Our sodliers would be more appreciated than ever, for a harder country to march over and fight for there cannot be; and beyond Ladysmith it is almost as bad. Newcastle bids fair to be a popular station with the men. We have only pitched some of our tents, as we are expecting new E.P. tents any minute. We have just opened one hospital, and have taken in 120 men and 12 officers. Although we are still sleeping under canvas, we have a small house for messing and storing, and therefore feel quite settled down."

### MIDDLESBROUGH NURSING ASSOCIATION.

The Lady Superintendent of the Middlesbrough Nursing Association sends us the daintily got-up report of that organisation, and of the Middlesbrough Children's Fresh Air Fund. The association has now been in existence for ten years, and, as the report says, can look back upon a decade of useful and ever increasing work. That it has a strong claim for support on every class of society in the town is shown by the figures—in 1899 the lady superintendent of the district nurses had 780 cases and paid 21,171 visits—but we are sorry to see that the claim is not recognised as it should be. For the last three years the expenditure has exceeded the income, "and the committee feel that they may have to decrease the number of nurses, and thus lessen the usefulness of the association." As the staff only consists of four nurses, they should obviously be rather increased than diminished in numbers. The Children's Fresh Air Fund has been established for upwards of four years, during which time 69 children have been sent away every alternate Tuesday between May and September for a fortnight to the seaside or the moors.

### THE PRINCESS MARY VILLAGE HOMES.

An appeal is made at the right moment for help towards the erection of a permanent cottage in connection with the Princess Mary Village Homes at Addlestone, for it is at this season of the year that the thoughts of children turn longingly to the seaside. The trustees do, indeed, rent a cottage in the summer months for the use of the children, but it would be a great advantage if they had one of their own. We hope that the good work done by the homes during a period of nearly thirty years may speedily enlist sufficient public support to secure the thousand pounds which its managers require in order to purchase one.

### A VIVANDIÈRE.

"The other day," says a correspondent, "whilst being shown over a large hospital in Austria, I noticed a curious incident. In one of the men's wards a nurse was carrying under her arm a small wooden barrel. Presently I saw her dole out to several patients a glass of wine. It seemed such a funny way of giving stimulants in a hospital. The nurse looked like a *vivandière*."

### SHORT ITEMS.

A communication has been received by the Lord Lieutenant of Ireland from Princess Christian requesting that it should be made known at once that she is asking for recruits for the Army Nursing Reserve Service. Her Royal Highness would be glad if any Irish nurses who would be willing to volunteer for such service would send in their names without delay to Colonel Johnson, Army Nursing Reserve Service, 18, Victoria Street, S.W., who will furnish all particulars. —The fancy fair held at the Devonshire Park, Eastbourne, last week, resulted in the funds of the Princess Alice Memorial Hospital being benefited to the extent of nearly £1,000.—An excursion to Walton-on-the-Naze, in aid of the funds of the London and District Poor Law Officers' Association, will take place on Friday, July 20th. Tickets can be obtained at the offices, Tooley Street, S.E.—The annual meeting of the Maternity Society will take place on Thursday, the 12th inst., at the Salle Erard (Great Marlborough Street), at seven p.m., Dr. Josiah Oldfield in the chair. It will be followed at eight p.m. by a conversazione and soirée musicale in aid of the funds of the charity, under the patronage of the Countess Cadogan. Tickets, 5s. each, can be obtained of the secretary at the Maternity Home, 145, County Terrace Street, New Kent Road, S.E.

### Lectures on Nursing for Probationers.

By E. MacDowel Cosgrave, M.D., &c., Lecturer to the Dublin Metropolitan Technical School for Nurses.

### X.—THE CIRCULATION.

As the tissues of the body are continually wearing, and as oxidation or burning takes place to keep up the temperature of the body, some provision must be made for fuel and repair. This is done by the blood, which carries nourishment in its fluid part and oxygen in its red corpuscles, delivering them

Fig. 17.—The Interior of the Left Side of the Heart.

*LA*, left auricle; *LV*, left ventricle; *ao*, aorta; *pa*, pulmonary artery; *mv*, mitral valve; *sv*, semilunar valves of aorta; *sv'*, the same of pulmonary artery.

throughout the body and at the same time taking up and removing the waste. To carry out its functions the blood must first pass through the digestive organs and lungs so as to gather up supplies, through all the living organs and tissues to give them what they require and to receive their worn-out materials, and through the excretory organs in order to get rid of these waste matters. The blood passes through three classes of vessels. It leaves the left side of the heart by the large artery—the aorta—which gives off branches that, like itself, have strong elastic walls whose contractions help to pass on the blood; these arteries give off other branches and get smaller and smaller until they can no longer be seen by the unaided eye. On its way back to the heart the blood passes through veins; these begin as small vessels which gradually run together, getting larger and larger until they form the two large vessels, the superior and inferior vena cava, which pour the blood into the right side of the heart; the veins are more numerous than arteries and have weaker, softer walls.

Between the ends of the smallest arteries and the commencement of the smallest veins are the third class of blood-vessel, the capillaries. These are so small that they require a powerful microscope to render them visible, and whilst the arteries and veins are only used for the conveyance of blood, the capillaries have such thin walls that the fluid of the blood containing nourishment and oxygen can easily pass out into the tissues, and the fluids of the tissues with used materials and carbonic acid gas can pass into the capillaries.

The organ that causes the blood to circulate through the vessels is the heart; being made up of muscle it can contract, thus squeezing the blood out of its chambers. The two sides of the heart act simultaneously, the weaker right side sending the blood up to the lungs for purification, the stronger left side sending the pure blood throughout the entire body. Each side of the heart has two chambers, an auricle in which the blood collects, and a ventricle, which drives out the blood supplied to it by the auricle, so the auricle may be looked upon as the collecting chamber, the ventricle as the force pump.

The blood can only flow in the proper direction, as there are valves at the entrance and exit of each ventricle; these valves are folds of the lining membrane of the heart, and are so arranged as to be washed back against the walls when the blood is flowing in the right direction, but if it moves in the wrong direction it washes them out, so that they meet in the centre and stop the way.

The heart beats some seventy or eighty times a minute. First the auricles contract, driving the blood into the ventricles; then the ventricles contract, driving the blood into the arteries; then there is a pause, during which the auricles are refilled. For each beat of the heart this sequence is repeated.

The circulation can be divided into two parts; in each the blood leaves a ventricle and returns to the auricle of the opposite side. The lesser, or pulmonary circulation, starts from the right ventricle, passes through the lungs, where the blood is purified, and ends at the left auricle. The greater,

Fig. 18.—The Circulation of the Blood.

or systemic circulation, starts from the left ventricle, passes through all the organs and tissues of the body, and ends at the right auricle. The course of the circulation is, therefore, from the right auricle to the right ventricle, from that into the pulmonary artery, which conveys it to the lungs, where it goes through countless myriads of capillaries; then through veins, which finally join into four large pulmonary veins opening into the left auricle. From the left auricle into the left ventricle, from that into the aorta artery, thence to the arteries, capillaries, and veins of the head, trunk, and limbs, being finally gathered into the two large vessels, the superior and inferior vena cava, which open into the right auricle.

A third part may also be described, this is called the portal circulation. The blood from the digestive canal is carried by the portal vein to the liver; here the vessels break once more into capillaries, which re-unite, forming the hepatic veins. So, in this circulation, the blood passes through two sets of capillaries—those in the walls of the intestines, and those in the liver—before returning to the heart.

If there is difficulty in forcing the blood through the lungs the right side of the heart suffers; if there is difficulty in forcing the blood through the systemic circulation the left side suffers. If the difficulty comes on suddenly the heart gets dilated and weakened; if the difficulty comes on slowly the muscular fibres increase and the walls of the heart get thicker and stronger. When more blood is wanted in any part the blood-vessels dilate; if necessary the action of the heart quickens; thus in digestion blood flows to the walls of the stomach, &c.

The action of the heart can be affected by treatment in three ways: (1) It can be made quicker, most so-called stimulants have this effect, they also dilate the blood vessels; heat, alcohol, ammonia, and ether have this effect, so also have all diaphoretics—medicines which cause perspiration. (2) It can be made slower; cold, digitalis, strophanthus, aconite, and hydrocyanic acid all have this sedative effect. (3) It can be made stronger; digitalis, strophanthus, strychnine, caffeine, and iron all have a tonic effect on the heart.

### Interview with Mr. Burdett-Coutts, M.P.

By Our Commissioner.

One result of the serious charges made by Mr. Burdett-Coutts, M.P., against the War Office respecting the treatment of our sick and wounded soldiers in South Africa is that the member for Westminster has ever since they became public property been overwhelmed by correspondence, and it was not until Tuesday morning that he was able to keep an appointment with me. As I explained at the outset, I did not desire to discuss the general points raised by him, but to obtain from him any information, and the expression of any opinions which might be of special interest to the nursing world. Obviously, however, the nursing is the essence of the question, and perhaps it is not too much to say that Mr. Burdett-Coutts puts it first of all.

### Hostility to Female Nursing.

"In one of my earliest articles," observed the member for Westminster, "I drew attention to the hostility of the Army Medical Department to female nursing. The traditions of the Department have always been against it, and the requests of the private hospitals for more nurses than the regulations of the Department allowed were persistently refused."

"What did the regulations allow?"

"In a general hospital eight nurses and one superintendent to every 520 patients, an allowance which means that the female nurses are not to do any nursing but are simply employed to superintend orderlies."

### The Yeomanry Hospital.

"After considerable effort a concession was made, and the number of nurses for 105 patients was increased to four. Then the Yeomanry Hospital came along, and, thanks to social influence and to the determination of Mr. Fripp, forty nurses were permitted to minister to the necessities of the patients."

"Do you care to express any opinion about the Yeomanry Hospital?"

"I think it is a splendid institution, and that the greatest possible credit is due to Lady Curzon and Lady Chesham for organising the fund; to Mr. Fripp for organising the hospital, and to Colonel Sloggett, R.A.M.C., for the skilful manner in which he 'commands' it."

### Lord Iveagh Denied Nurses.

"Another illustration," continued Mr. Burdett-Coutts, "of the antipathy of the Department was shown in the case of the Irish Hospital organised by Lord Iveagh at the same time as the general hospitals. They made strong efforts to secure permission for the employment of female nurses, but could not obtain it, and after the hospital arrived at the front the managers had to take what nurses they could get. The fact is that the Department would not face the necessity for the appointment of female nurses, and, as I have stated in one of my articles in the *Times*, the absence or totally inadequate supply of female nursing laid down in the manual of the Department is a glaring blot on our present Army Medical system. It is, I repeat, an antediluvian prejudice dating from the time when 'Mother Gamp' ruled in the sick room, and taking no account of the enormous development of scientific and efficient female nursing which has been one of the brightest features in the domestic history of the last thirty years."

### The Arguments Against Female Nursing.

"It would be interesting if you would indicate the arguments that are used against female nursing in the army in time of war, and your own answers."

"I have already done so at some length; but, possibly, a summary may suffice for the purpose. It is urged that soldiers prefer to be nursed by orderlies, and do not like women about them when sick or wounded."

### The Ministering Angel.

"My reply is a direct negative. Any man who has been seriously ill knows the difference between an orderly with horny hands and creaking boots, smelling of tobacco and other things, moving about his bed, tending him with a man's touch, and the real ministering angel—the female nurse. Then, it is contended that a hospital camp is not a place for women to live in. I maintain that it should be made a place, and that this has been done repeatedly with perfect comfort and propriety."

"You have disposed of two of the objections; are there any others?"

"It is alleged that as a number of the patients in a military hospital are convalescent, and sit about smoking and chatting, a woman's presence interferes with their freedom and enjoyment of each other's society. Even if this were the case, however, it would only prove that military hospitals ought not to be occupied by convalescents. But the general experience is that the presence of a woman invariably raises the whole tone of a hospital. Another contention is that all cases are not suited to female nursing. My rejoinder is that this is merely a question of classification and separation, just as easy to accomplish as the isolation of enteric or scarlet fever cases.

### The Sentimental Difficulty.

"There is yet one further objection, which I may call the sentimental difficulty. But there is no more danger of flirtation in the ward of a military hospital than in the male ward of a civil hospital. To put forward such an argument against the employment of female nurses in civil hospitals would be to excite an outburst of ridicule."

"And you think that arguments of the character you have described have had a great deal to do with the indisposition of the authorities to send a requisite number of female nurses to the front?"

"I can only say that they are the arguments which I have frequently heard used, although I should not like to affirm that they are endorsed by all the officers of the R A.M.C. On the contrary, I know that some of the officers cordially welcome female nursing. But this does not alter the fact that at the head-quarters of the department at home the prejudice is as strong as ever.

### The Bloemfontein Hospitals.

"To the existence of this prejudice I mainly attribute the state of affairs which I found at Bloemfontein on May 23rd. Although we had then been for ten weeks in possession of the town there were in one hospital only twenty nurses for 1,700 patients. I consider that this is a wretched number, especially bearing in mind the lesson taught us by Florence Nightingale at the Crimea, and that the present war is carried on in a safe country for women."

In connection with Bloemfontein, Mr. Burdett-Coutts pointed out that he gave a favourable account of the town hospitals, and I asked him how many patients were in them?

"There were," he replied, "eight such hospitals, with an aggregate of about 750 patients. In three of these there were nurses or sisters on the arrival of the troops at Bloemfontein. They, of course, cenfined themselves to their respective institutions. These contained about 260 patients —sixty in the Roman Catholic Convent, a hundred in St. Michael's Home, an Anglican institution, and a hundred in Volk's Hospital. In all these cases the patients were well nursed, and Miss Mabel Young, the matron of Volk's Hospital, was indefatigable in her attendance to their needs."

### Complaints from Nurses.

"Since your article which caused such a stir appeared, have you had any letters from nurses?"

"A large number, many of them complaining that the writers were rejected when they offered themselves for employment at the front. Of course, I know that some applications of the kind were properly refused; but I have had four or five letters from fully-qualified nurses who were apparently rejected simply because they were 36 instead of 35 years of age. But the truth is that the objections of the Army Medical Department to female nursing have permeated the very organisation which was started to encourage it."

"You mean the Army Nursing Service Reserve?"

### Red Tape in the Reserve.

"Yes, unfortunately; the Department has introduced even there the red tape and restrictive regulations which militate so greatly against female nursing. It is a striking commentary on the policy pursued that, owing to the Department not having sent out sufficient female nurses from home, they have had to take on a large number out there."

"Do you know whether the latter were qualified?"

"I cannot tell you; but it would obviously be better to avoid any risks of having to fall back upon untrained or inadequately trained nurses by sending from here a sufficient quantity of those who are known to be properly qualified."

### The Crux of the Question.

"How many nurses do you consider ought to be employed at the front?"

Mr. Burdett-Coutts modestly rejoined: "That is a subject on which the profession is more competent to judge than I am. But my whole position is that if in the general hospitals they had a proper elasticity in their system they could have turned all the base hospitals at Cape Town into hospitals served by women, and then despatched to the front the whole of the orderlies and male staff, with the exception of a few of the latter to maintain discipline and nurse unsuitable cases, and a certain proportion of untrained orderlies, even privates in ordinary regiments, to do the heavy work. It was because of the insufficient supply of female nurses that such a large male staff had to be kept at the base, when their proper place was elsewhere. These men should have been attending to the sick and wounded at the front in dangerous places, instead of registering all the details of wounds and diseases in the office of a base hospital, or doing women's work in the wards."

"You do not deny that men are wanted to do the heavy part of the work?"

"On the contrary I consider it is essential that they should. But the absurd thing is that in addition to the nursing orderlies there are outside men—Bât-men they are called—to do the heavy work. This shows that the trained orderlies are employed exclusively as nurses, and that the theory of the system is to have the nursing done by males."

### Misrepresentations.

In conclusion, Mr. Burdett Coutts mentioned some of the misrepresentations to which he had been subjected, and said, "In the first place I have never attacked the *personnel* of the R.A.M.C.; in the second, I have never attacked, nor had the slightest intention of attacking, Lord Roberts. My sole object has been to show what has happened in South Africa, in order that reform may come, and that such deplorable things may not occur again."

### Lord Wolseley's Views.

"You wish to change the system?"

"Exactly. But you may judge how hard that task is likely to be when I state that Lord Wolseley is very strongly in favour of female nursing in military hospitals."

Certainly the fact that the views of the Commander-in-Chief of the Army upon nursing are flouted by the War Office authorities will tend to materially confirm the impression that the permanent officials of the Department—like some well-known individuals centuries ago—take too much upon themselves, and will have to be either taught their place, or relieved from the performance of duties which they misinterpret.

### Nurses' Reading Society.

Miss Moberley, 24, Portland Place, London, W., sends a short statement of the accounts of the Nurses' Reading Society at the end of its first year, July 1st, 1899, to July 1st, 1900:—

Receipts: Subscriptions—July, 1899, £4 15s.; October, 1899, £3 5s.; January, 1900, 12s. 6d.; total, £8 12s. 6d. Expenses: Postage and cyclostyling, £1 4s. 8d.; money set apart for prizes, £2; secretarial expenses, correcting papers, stationery, £5 7s. 10d.; total, £8 12s. 6d.

When the examination papers for last quarter have been sent in and corrected, a list of the marks obtained and the names of the prize winners will be published.

Mr. Glaisher, 57, Wigmore Street, who arranged a library in connection with the society, will sell books that have been used by members at a reduced price after they have been lent in the library.

### Nursing in America.

By an English Nurse.

IV.

I have said that my patients were of all classes—I ought to have said there were rich and poor, for up there, buried away among the mountains, I found equality and fraternity the vogue. Colonel Brown's wife, who paid £5 per week for her private room, spent hours in the company of the little laundry girl paid for by the city. The Hon. Mr. Jones was only too glad to get the teamster with the broken ankle to come and play cribbage or euchre with him. There were no servants—only helps—who shared, as a matter of course, in the general life of the family, and, unlike the people whom I had known in the cities, they were pro-Boer in their sympathies to a man.

I had got among a different type of New Englander altogether, among the men who chewed and expectorated freely and said "darn" and "guess," and the women who had worked for their school and college expenses, and had taught at school, and then married and had children, and had toiled and slaved to help make money and bring them up, who washed and baked, and kept cows and poultry all unaided, and generally wore their lives away for the sake of their families long after it had ceased to be necessary for pecuniary reasons. But from these, as from their more polished city brethren, I met with nothing but kindness and courtesy. Just as soon as they knew I was English and where my sympathies were, they left off abusing England, at any rate in my hearing. And when a Boer victory was announced in large type they never asked if I had seen the local papers, whereas if the smallest English success were reported I was greeted with a chorus of "Good news for you to-day, Miss!" and when I said once I was afraid good news for me meant bad news for them, they answered stoutly, "Darn it! What is it to us compared to what it is to you? Bless you, we don't really care! Let the best man win is our sentiments, and perhaps the Boer ain't the best man after all."

### Day Duty.

At the end of five weeks, spent happily enough, the matron asked me if I would mind coming on day duty to fill a nurse's place who was leaving, and to make room for an older sister of her own who would take night, but did not feel equal to day, duty. I did mind very much, for I was pretty sure I could not stand day duty under the conditions which then prevailed, for everything was in a state of chaos. The matron was herself new—the entire previous staff had resigned with the former matron, and had been departing one by one. The "Training School," for it had professed to give some twenty young women a two years' thorough hospital training, had been broken up. Expenses had been cut down to the lowest possible point, and the new matron was wearing herself into a fever endeavouring, with a reduced and insufficient staff, to evolve order out of chaos and to restore the credit of the place, which had suffered somewhat severely under the former *régime*—she herself having no further experience save her three years' training and a few months' experience as a night superintendent to go upon. However, as I only intended to remain two or three months at most it would have been churlish on my part to raise objections, so I agreed with inward unwillingness, but as good an outward grace as I could command.

### The Hours on Duty.

I had found when I was on night duty that by going to bed as soon as I came off duty and rising and going out in the afternoon I could keep pretty well, in spite of the fact that 75 deg. was the regulation heat, but on day duty there was no time for that, and I soon began to suffer from lack of fresh air. The official day began at twenty minutes to seven, when the breakfast bell rang. At seven we went to our wards and got our patients' breakfasts, after that the usual routine of work went on; there were dressings to prepare for surgeons; douches to give (I had eight cases of curetting during my three weeks on day duty); beds to make; massage to be given; lunches to prepare—in fact, the usual day's work of the average hospital. In addition to this, as the cook only prepared full diet, when you had patients on milk diet or with delicate appetites, one had to forage and concoct dainty dishes as best one could from such materials as one could command.

### Work Harder than in England.

I found the work much harder than I had ever found it in England—it seemed to me there was so much waiting upon the patients to be done, and so little real nursing. Medicines were omitted as often as they were given. Few temperatures were taken. Nearly every patient had a different doctor, and these came and went at all times and seasons, sometimes giving their orders to the nurse, at others to the patient. So things got mixed, and instead of having the doctor's orders clearly written out on the bed tickets as is customary here, the doctor simply said what he wished done or administered, the nurse making a note of it in her note-book. A stock cupboard of medicines was kept, and nearly all prescriptions were made up by the nurses in the hospital.

On the other hand, the doctor expected the nurse to keep copious notes of each case, these notes being written out and hung over the bed ready for his perusal, so that if the nurse were not there he could see exactly what his patient had done and taken since his last call.

### The Trying Trays.

I think I found the trays the most trying. Each patient, paying or otherwise, had his or her own tray, cruet, sugar basin, cream jug, glass, &c. As far as serving the food went it was done very daintily, each nurse taking pride in always having clean tray cloths and serviettes (to accomplish this frequent raids on the laundry were necessary, and it was a case of each for herself). No nurse would think of taking even a glass of milk to a patient without a tray, covered with dainty cloth, but, on the other hand, so much time was frequently spent on getting the trays ready that the food was as often as not cold, the tea stewed. These trays were "set" in the diet kitchen, of which there were two, one at each end of the building. We had then to carry them to the central or main kitchen, where the food was put on the plates, the cups or glasses filled up, then we carried them back to the patients. When one came to have eight or nine trays thus to prepare and carry, without being accustomed to such work, meal times were something to be dreaded, and, as I often did "relieving duty," I frequently had thirteen or fourteen.

### The Diet.

The three principal meals of the day were pretty much alike in character. At each, tea or coffee was served and meat and various vegetables provided. The patients' dinner was at twelve, the nurses' (off the same joints or dishes) at half-past twelve. It was not much appetite that most of us brought to the table after watching the cook cut off helping after helping, and patient after patient turn it over on their plates, as it is the way of patients to do. Iced water was drunk with everything and at all times. To pneumonias and those patients with high temperatures ice cream was given freely, and seemed to suit.

### The Sick and Wounded on the "Montfort."

### A CHAT WITH ONE OF THE NURSES IN CHARGE.

By a Correspondent.

"I would gladly have stayed in Johannesburg," said Sister Kathleen, who had just arrived in England in part charge of the sick and wounded on board the transport "Montfort," "but we were all turned out at 10 hours' notice, 10 days after war was declared."

"You would have stayed to nurse the Boers?" I suggested.

"Yes; but the doctor—a German—who was put in charge when the hospital was taken over by the Government for military purposes, seemed to think we might poison his patients, and we all had to go, including the matron, though we had given our word of honour to remain until it was all over. Of course, a patient is a patient, whether English or Dutch, and we should have nursed the Boers just as carefully as any other patients."

"'What are you going to do,' we asked him, 'without any nurses?' To which he replied that he had arranged with some ladies—friends of his—to come in and nurse!"

### Sent Adrift.

"It must have been very awkward to be turned out," I said, "with no time to make arrangements where to go?"

"It was, extremely awkward, especially as our friends in the town had left. My parents had been gone a month; and the worst of it was that all of our most valued possessions, including a piano and some pictures, of which my mother was very fond, had been left in my care at the hospital. There was no time to move them, and I don't expect ever to see them again."

"And did your parents go to Cape Town?"

"No; they went to a quiet little place near Grahamstown, where they have been quite undisturbed, though, of course, they have been wretched, in suspense as to the fate of our home in Johannesburg, and with nothing to do."

"And what became of all the nurses?"

"The people in the town collected money for us. Some came to England at once, and I have since heard that one has been made head of a hospital in Dublin, so I suppose she will not return. I volunteered for the front, but was not accepted. I was told that a number of nurses were coming from England, and that they could not be disappointed. However, the authorities took my name and address with those of the others who volunteered, and we were informed that if we were required we should hear later."

### Interrupted Training.

"Some of the nurses," Sister Kathleen went on, "had only lately joined, and others still wanted some months to finish their training. I myself was not fully trained, and I am very anxious to get back, as I want my three years' certificate."

"Have you had any military nursing," I asked, "between leaving Johannesburg and coming over on the transport?"

"No. I was ill at home for some time; then I had a typhoid (private) patient for two months, and then the order came for me to come out."

"That is, 'home,'" I said, smiling.

Sister Kathleen is not the first colonial nurse to whom I have spoken who feels it rather hard that more use was not made at the outset of the war of the colonials on the spot, ready and waiting for the military nursing experience, and well acquainted with the conditions, climatic and otherwise, of the country.

"Perhaps," I suggested, "the War Office knew that there are colonial nurses who are by no means a credit to the profession, and thinks they are all alike?"

"They would have found plenty to choose from in the Johannesburg Hospital," she replied, "and we should have been thankful for the experience it would have given us. However, I am very glad to have been sent on the transport. The change from hospital was very great. To begin with, of course I had only been 'Nurse' before, and it took me some time to get used to hearing myself spoken to as 'Sister.' When I went on board the medical officer in charge told me that there were over 290 patients (I forget the exact number, but it was not much under 300), and I made up my mind at once that we should never reach England alive, for there was only one other nurse besides myself, Sister Bolton. But, as a matter of fact, there was less work than in hospital; at any rate, for the first few days. Sister Bolton took the night work, and I did day duty."

### The Enteric Patients.

"I suppose most of the patients were suffering from enteric?" I inquired.

"Yes, nearly all. But they were convalescents chiefly, and we had only two cases that gave us great anxiety. Even the orderlies were convalescents, and so they were not able to do as much to help as ordinarily, but they did all they could, and gave as much assistance as we required. But you can't imagine what it felt like to have to walk on dirty floors all day. The edges of my skirts were black in a very short time, and I required three aprons a day. The boat was really a cattle boat, and, although it was said to have been cleaned for us, it was the dirtiest place I have ever worked in."

"And what arrangements were made about beds?"

"The men slung their hammocks up in the hatches ordinarily used for the cattle. I had seven dresses in the three weeks, and you can imagine how I felt, arriving in England with my boxes full of dirty clothes."

To my inquiries as to food, Sister Kathleen replied that this was excellent, both for the staff and for the patients. "The only thing was that, of course, we could not have fresh milk; but sterilised and condensed did quite well."

### Officers versus Soldiers.

"Had you much to do with the officers?" I asked, recollecting what another nurse had told me, namely, that she much preferred to nurse private soldiers. I found that Sister Kathleen was of the same opinion.

"I very much prefer the Tommies," she said. "They are most grateful for what you do for them, and they don't take your attentions as if they had a right to them. Some of the officers were extremely nice, but others had a good deal of 'side.' But we had not much to do for them; I did a dressing every day for a gunshot wound in the lung, but most of them had their servants with them, so we had nothing to do with keeping their rooms clean, &c. One had sciatica, and had to have a soda bath every day, but he had his servant with him."

"Have you seen anything of the plan of inoculation for enteric?" I asked.

"No; but the doctor told me that he had inoculated a good many men on the way out with great success."

"And about the two who were so ill?"

"Yes, I was going to say, we were so thankful not to lose any of our men; it must be terrible to have to leave them at sea. One was a great anxiety, and we never left him, after he had a relapse, night or day. The relapse was brought about by his obtaining a piece of meat from a friend, and eating it while I was absent for certainly not more than three minutes; I think I had gone to fetch a temperature chart. Of course, he became rapidly worse, his temperature went up to 105, and it was only by repeated doses of champagne that we kept him alive. We thought he would have died before reaching Cape St. Vincent, but he pulled through."

"What became of the men when you landed?"

"We left most of them at Plymouth, but some came on to Woolwich; they were all able to walk except these two serious cases."

"Were you ill at all on the voyage?" I asked.

"Not in the least, and my appetite was very large, I think as large as the chaplain's. He, too, was recovering from enteric, and he required food every few hours, breakfast at half-past eight, wine and biscuits at ten, more food at twelve, lunch at one; mere food at three, tea at four, and dinner at half-past six."

Nearly Washed Away.

"Now," I said, "I am going to ask what you have heard of the complaints that are filling the papers just now about the nursing arrangements?"

"I have not seen anything myself," Sister Kathleen answered, "and all I can say is that both men and officers have spoken very highly of the hospital arrangements at Wynberg; in fact, they did not seem able to say enough in praise of the treatment there."

"Did you see anything of the ladies whom everyone now calls 'silly women'?"

"No, but one of my patients told me he had been washed so many times that he wondered he was not entirely washed away. And as for eau de Cologne, he had had so much on his forehead that he was perfectly sick of the smell!"

Finally, I learnt that Sister Kathleen had only a few more days in England, as she had requested to be sent back as soon as possible. She had landed in a pitiless downpour of rain, had been disappointed to find that friends whom she hoped to meet had gone back to the Cape, and would immediately have gone back had there been a boat available.

"But now," she said, "I have made many friends, and you can imagine how sorry I am to be going so soon. However, I shall ask to be sent over again, and hope to spend a longer time here then."

### Minor Appointments.

Birmingham General Hospital.—Miss Ada S. Burgin has been appointed Night Superintendent. She was trained for three years at Guy's Hospital, and has since been night superintendent at East Dulwich Infirmary, matron of the Woodstock Hospital, South Africa, and midwife at the Fulham Nursing Home.

St. Saviour's Infirmary, East Dulwich Grove.—Miss Mary Cozens has been appointed Sister. She was trained at Kensington Infirmary, and has been sister for two years in that institution, as well as charge nurse for a year at the South-Western Hospital.

Portsmouth Workhouse Infirmary, Milton.—Miss Jane Mackay has been appointed Charge Nurse. She was trained at the Children's Hospital, Great Ormond Street, and has since been nurse at St. Olave's Union Infirmary, and the Guildford County Hospital.

Cumberland Infirmary, Carlisle.—Miss Mary E. Bennett has been appointed Staff Nurse. She was trained at Meath Hospital and County Dublin Infirmary. She has since been staff nurse at the City Fever Hospital, Old Swan, Liverpool.

Wheatenhurst Union.—Miss Alice Greenhalgh has been appointed Assistant-Nurse. She was trained by the Meath Workhouse Nursing Association, at Blenheim House, Kew Gardens.

Ecclesall Bierlow Workhouse Hospital.—Miss Mary J. Garratt has been appointed Charge Nurse. She was trained at Guy's Hospital, and has since done private nursing.

Children's Hospital, Bradford.—Miss Jane Gregory has been appointed Staff Nurse. She was trained for three years at the Llandudno Cottage Hospital.

### Queen Victoria's Jubilee Institute for Nurses.

Her Majesty has been graciously pleased to approve the appointment of the following as "Queen's Nurses," to date July 1st, 1900:—

England.—Susan Cottrell, Rose A. Clark, Elizabeth M. Thaw, Constance A. Haynes, Agnes Beer, Amy F. Toole, Elizabeth Pople, Clara E. Busselle, Emma Greensill, and Mildred Purcell, London; Christian Bell and Annie Quinn, Liverpool; Ada M. P. Ashworth, Laura Hill, and Mary A. Price, Salford; Anna E. Peltzer, Ellen Goble, Ethel Raymond, and Annie Harrison, Manchester; Kate E. Barlow, Leeds; Annie M. Chandler, Rochdale; Mary G. Milne and Jane Bell, Gateshead; Alice A. Baumgarton, Malvern; Mathilde Bull, Birkenhead; Lucy E. Lathlean, Wisbech; Mary J. Scott, Neston; Kate Parker, Ellel; Louise Boulding, Haydock; Norah O. Greene, Bingley; Isabel A. Driver and Ada Boulding, Haslingdon; Alice J. Maclachlan, Pleasley Vale; Annie H. Morrison, Bedworth; Maud M. Long, Bedford; Mary Jones and Kate Allin, Gloucester; Maud Fletcher, Bath; Elizabeth Marsland, Huntingdon; Kate Wilkinson, Cheltenham; Helen Ellis, Shelford; Hilda Carrington, Chatham; Caroline M. Robinson, Strood; Mary E. Taylor, Southampton; Lily Dodds, Gosport; Louisa J. Lawrence, Snodland; Ellen Parnell, Little Berkhamstead; Edith S. Pryse, Chard; Katherine Macdonald and Mary M. Heddle, Torquay; Emma Clarke, Looe; Marion A. Webb and Minnie Anderson, Penzance; Muriel H. Powell, Northampton.

Wales.—Mary E. Evans, Bangor; Ann C. Roberts, Bethesda; Alice M. M. Corns, Dolgelly; Jane M. Price, Cefn; Eleanor D. Hitchings, Llandudno; Harriet L. Edwards, Llanrwst; Myfanwy Davies, Amlwch; Sara L. Crowther, Nantlle Vale; Rachel Davis and Sarah M. Jenkins, Neath; Mary G. Alford, Merthyr Vale; and Mary Williams, Pontypridd.

Scotland.—Elizabeth Small, Margaret Sandison, Agnes Wasson, Jessie McKinnon, Helen Kerr, Helen Hogg, Margaret M. White, and Elizabeth H. Robertson, Edinburgh; Jane Allen, Whifflet; Catherine A. Chisholm, Margaret T. Wallace, and Mary J. Chappelow, Glasgow; Isabella N. Blackwood, Dundee; Margaret B. Smith, Paisley; Mary A. Morrice and Margaret L. Gill, Coatbridge; Grace Rigby, Lanark; Sarah E. Clarke, Largs; Sarah F. King, Port Glasgow; Letitia Bennett, Hamilton; Matilda Ormiston, Brechin; Ellen C. Crichton, Aberlour; Ellen A. Garden, Thurso; Eliza MacPherson, Otter Ferry; and Aleina Maclean, Kilfinechan, Ross of Mull.

Ireland.—Mary Givens, Ada L. Borlase, Frances G. Love, and Aileen O'Halloran, Dublin; Louisa L. Butler, Strabane; Sophia Murless, Killarney; Minnie Blackburn, Hillsborough; and Jane F. Hennessey, Swords.

### Appointments.

Hampstead Nursing Association.—Miss Susan Maria Masters has been appointed Superintendent. She was trained for three years at the Norfolk and Norwich Hospital. She has since been staff nurse at the same institution, on the private nursing staff of the hospital, matron of the Mildenhall Cottage Hospital, and district nurse in connection with the North London Nursing Association.

Maud Hospital, Exmouth.—Miss H. Marks has been appointed Matron. She was trained at the Devon and Exeter Hospital, where she remained for six years. She has been sister-in-charge at the Maud Hospital for the last three and a-half years.

Darwen Isolation Hospital.—Miss Plough has been appointed Matron. She was trained at the Royal Surrey County Hospital, Guildford, and has since been matron at the Sanitary Hospital, Leighton Buzzard.

### Echoes from the Outside World.

### AN OPEN LETTER TO A HOSPITAL NURSE.

SINCE writing last week there has been much to sadden us. It is true that Admiral Seymour and his brave little force are safe, but every day shows more and more the magnitude of the rising in China, and the grievous trouble which is in front of us. According to report, Prince Tuan has usurped the Imperial power, and ordered all the viceroys and governors in China to drive the foreigners into the sea, and the Emperor and the Dowager have fled. The message from Sir Robert Hart which was despatched from Pekin as long ago as Sunday week by a Chinese messenger, though only reaching Tientsin many days later, was heart-breaking. "The foreign colony is besieged in the Legations. The situation is desperate. Hasten." And then to learn that the Admirals have decided that the relief of Pekin is not at present possible! The German Minister had been murdered prior to the despatch, and it is feared was only the first of many who will lose their lives at the hands of the Chinese rebels. But till our worst apprehensions are confirmed, we can at least hope. Whilst the sword was slaying in Asia, in North America fire was devouring men, women, and little children within sight of their fellows, who were powerless to attempt a rescue. The scenes at the fire at the Hoboken Docks, New York, on Saturday afternoon were almost unparalleled in their horror. Not only the sailors, but those sightseers who had come to inspect the large steamers, were caught as in a trap below the decks, and were unable to get out, whilst the flames crept nearer and nearer. With their heads out of the portholes they besought the onlookers to help, and besought in vain, for none could break the steel plates which lined the sides of the vessels. Once or twice a poor frantic mother threw her baby out of the hole through which she could not pass herself, and it fell into the water. At any rate, the hapless infant was spared the agony of being burnt to death, though the chance of rescue was small. The Hamburg-American Company blew up their pier with dynamite, thus preventing the flames from spreading to other docks, and by bravery and presence of mind Captain Englehardt saved the "Kaiser Wilhelm der Grosse," but the loss of life and of money is already known to be fearful, although it will take some time to ascertain the exact state of the case.

THE visit of the Khedive terminated on Wednesday, and in a week he managed to get through practically the whole of the programme arranged for him. Curiously enough, on the day of his arrival, when I was not thinking about him, and was not intending to make any special effort to see him during his brief stay here, I had occasion to walk into Charing Cross Station, and met him almost face to face. He is a pleasant-looking young fellow, and he showed no sign of recent illness either in his face or his gait. That he averted a serious attack by wisely giving himself up to treatment upon the first appearance of a bad throat is most probable, but his recovery was evidently complete, or he could not have worked as hard as he did while he was in London. His day begins so prematurely—half-past five seems to be his usual time for getting up—and I could not help wondering how the housemaids at Buckingham Palace liked him wandering about the corridors in those early hours which servants quite naturally look upon as their own "special," sacred to dusting and cleaning. He apparently was unacquainted with the European Royal custom—which always seems to me so comic—of returning a call within a few minutes of its being received; for after having paid a visit to Marlborough House, he came back to Buckingham Palace, and seemed—naturally—intensely surprised when the Prince and Princess arrived to see him a few moments later. At Windsor Castle he took an immense deal of interest in the Home Farm and the kennels, and said good-bye to the Queen in her breakfast tent at Frogmore. Before taking leave she presented him with the Grand Cross of the Victorian Order. He must have been much impressed on Tuesday with the enthusiasm of the English people, who remained standing in the streets throughout a thunderstorm so that they might cheer him as he passed. Had his visit to the City been in more propitious weather, he would have received a splendid ovation.

NEXT Monday, London's new railway—the Central—will be opened to the public, and should prove a boon not only to the business man whose "Time is money," but also to the nurse whose off-duty hours are short, and who frequently finds it difficult and sometimes impossible to see the friends or the "shows" at a distance and get back to her patients or her hospital in her limited leisure. The new railway traverses a district of the metropolis which has hitherto only been served by omnibuses and cabs, the former a slow, the latter an expensive, mode of transit. It is calculated that the fare by hansom from one end of the Central Railway to the other would be about 3s. 6d.—the train ticket will cost 2d. There is only one fare and only one class, but the carriages, which are open from end to end in American fashion, with arms to divide one seat from another, and brilliantly lighted by electricity, are very spacious and comfortable. From the Bank to Shepherd's Bush will take twenty-five minutes only, and trains will run every five minutes for a few weeks, and then every two and a half minutes. There is no dust, no dirt, and no sulphur fumes, notwithstanding that the lines are laid far down in the bowels of the earth, because electricity is the motive power. The "engine" does not look a bit like the steam locomotive we usually call by that name, but neither does the clean glittering building, lined with white glazed tiles, suggest an ordinary railway station. There are lifts to convey passengers from the booking offices to the platforms, which will probably be extensively used, but those who have an unconquerable objection to the feeling of dropping down, down, down, which is the peculiarity of a descending elevator, will find a staircase they can employ if they prefer it.

IT is not so many years ago that a bath-room in a house was looked upon as a distinct luxury, and a bath once a week was considered amply sufficient for the health of any ordinary individual, more especially if she were a woman. I believe that when Eaton Square was built not a single bath-room was provided for the tenants. But we have advanced a little since then, and it is a sign of the times that one of the French Ministers has provided the lady telephonists of the Central and Rue des Renaudes offices in Paris with hot and cold baths. It had been represented to him that constant telephoning was fearfully wearing to the nerves, and when he entered office he determined to find out for himself whether there was any truth in the assertion. He did not trust to his own judgment, but requested the post office doctor to accompany him, and, finding that all he had been told was quite true, he referred the subject to a Commission to see how matters could be remedied. The report of the Commissioners showed the desirability of shorter hours and the refreshment of bathing as a sedative for strained nerves. The former benefit seems more difficult to compass, but the latter boon has been granted, although, alas! not gratis. A bath, either hot or cold, costs three-halfpence, and a halfpenny more is charged for the towels. Later on efforts are to be made to provide the district post offices with the same boon. As yet I learn that no arrangements have been made at our own General Post Office to supply the female clerks with the means to obtain a bathe when over-fatigued, but it will probably come later. An interesting commentary upon this subject was curiously enough supplied recently in a letter from an army nurse at Bloemfontein.

### Everybody's Opinion.

[Correspondence on all subjects is invited, but we cannot in any way be responsible for the opinions expressed by our correspondents. No communication can be entertained if the name and address of the correspondent is not given, as a guarantee of good faith but not necessarily for publication, or unless one side of the paper only is written on.]

### THE ROYAL BRITISH NURSES' ASSOCIATION.

"A Matron" writes: I have a grumble against the R.B.N.A., of which I am a life member, in that I see, according to my request, Scotch midwifery certificates can also be added to any nurse's former list of certificates. But it is stated that all such must be paid for except L.O.S.! We in the provinces may well be without enthusiasm when we are so treated. Why should they make such a stipulation?

*** We referred the matter to the R.B.N.A., hoping that "A Matron" might possibly have been mistaken in her statement; but the answer of the Registrar is that "the L.O.S. certificate only is added to the members' lists of qualifications without any further fee."—Ed. *T. H.*

### OUR DUTY TO THE DYING.

"Another Nurse" writes: I am sure there are hundreds of nurses in the world who, with "Nurse H." have puzzled over the serious problem, "Our Duty to the Dying." Patients ought to know when their end is near, but I think when a doctor says a patient may linger on for some months harm is often done by telling this to the patient, especially if he be of a highly nervous character. A very helpful little manual has been published, called "Through the Long Night Watch." It is sold for 6d. at the Church Shop, Commercial Road, E. It contains several short passages suitable for saying by the bedside of the dying, besides several well-known Ancient and Modern hymns. "Nurse H." might find this book very useful to her.

### THE HOURS OF ASYLUM NURSES.

"A Nurse" writes: Kindly allow me a small space in your paper regarding the hours of asylum nurses. I work in a small private asylum where we take night duty in turns, and our hours are thirteen and a-half. We are supposed to have a monthly holiday, but often it is eight and nine weeks before we get it, and if we complain we are told that we are unreasonable. The matron thinks she is doing you a great favour by giving a monthly holiday. While on day duty our hours are from 6 a.m. till 9.30, with an evening off in the week from 6 till 9.30, and every Sunday morning and evening alternately. I should like to hear your opinion on this subject. If the Commissioners are shortening the hours in public institutions, they can surely do something for private ones.

### "THE OPERATION BASKET."

"Private Nurse" writes: I am very pleased to be able to tell "District Nurse" that I have used the "Operation Basket." The outside of the basket was covered with black waterproof, the inside had a detachable white linen lining. The contents of the basket I used were as follows: Two operating coats, two waterproof sheets, a horn case of sponges, a horn case of assorted drainage tubes, a syringe, a bottle of iodoform powder, a packet of iodoform plugging, a bottle of carbolic, the strength I forget, a bottle of corrosive sublimate tabloids, half a dozen bandages, two flannel binders, half a dozen wood wool pads, a roll of absorbent wool. Another basket contained what was required for the anæsthetic. What the charge was I am not aware, but if "District Nurse" wishes to know more about the "Operation Basket" she should write to Messrs. Davidson and Kay, chemists, Union Street, Aberdeen, N.B. They supplied the one I used. Since leaving Aberdeen I have never seen the "Operation Basket."

### ROMAN CATHOLIC NURSES.

"A Matron of a Maternity Charity" writes: I am glad to see that someone has the courage to take up the subject *re* Roman Catholics and general charities. I have often to appeal to the clergy of the various parishes in this city for extra nourishment for the poor women under our care, and in no case has help been refused to Jew and Gentile alike. But I am sorry I cannot say the same of the Roman Catholic clergy. It is either a vague promise which is never performed, or the cry "we are too poor." I ask, is it fair to let them shift their responsibilities on to other shoulders? All poor are dear to me irrespective of creed, and I think the Roman Catholic priest is apt to forget who it is who says, "Inasmuch as ye do it to the least of these My brethren, ye do it unto me." I hope the airing of this subject will stir up the Roman Catholics to a sense of their duty towards God's poor whom we have with us "always."

### A Day of Rest for the Metropolitan Nurses.

"On Wednesday last," writes a correspondent, "the usual annual opportunity was afforded the nurses of the London hospitals and Metropolitan nursing institutions of spending a day in the country at Hertingfordbury. This provision was made not only to afford a much-needed relaxation, but to help those who were present to realise in devotional exercises the spiritual character and dignity of the nurses' vocation as an essential part of the Christian ministry. The day commenced with a celebration of the Holy Communion in the parish church at eleven o'clock, the rector, Canon Burnside, being celebrant. The Dean of Windsor gave the address, taking for his text, 'Rest in the Lord,' and earnestly pointed out the necessity in an age of haste and worry of cultivating habits of quiet sustained communion with God, especially in relation to the peculiar strains and obligations of a nurse's life and work. Evening Prayer was said by the Rector at half-past three, the Dean again giving the address, based upon the Psalmist's words, 'Delight thyself in the Lord.' Allowing for all the difficulties of leaving their work a large number of nurses were present. They were entertained at dinner and tea, and occupied other intervals of the day in walking in the country. By the kindness of the Earl and Countess Cowper they were invited to visit the picture gallery at Panshanger. and this was greatly enjoyed. Before returning home they received some roses and bouquets of flowers, the gift of the Countess of Lytton, Mrs. Mackintosh, and Mrs. Leslie. The Rev. A. G. Locke, chaplain of St. George's Hospital, who kindly made the arrangements in London, was unavoidably absent."

### The Hanan Memorial Fund.

Major R. F. Ballantine, master of the Manchester Workhouse, Crumpsall, has received the following subscriptions towards the "Hanan Memorial Fund," viz.: Sister Dutton, £1; Sister Mackay, £1; Miss Haslitt, 10s.; Miss Guttridge, 2s. 6d.; Sister Beetham, 2s. 6d.; Nurse Waring, 2s. 6d.; Dr. Allan McDougal, £1 1s.; Nurse Mallinson, 10s.; Nurse Balkwell, 2s. 6d.; Nurse Jones, £1 1s.; Nurse Walker, 5s.; Nurse Shaw, 5s.; Nurse Parke, 2s. 6d.; Nurse Towle, 10s.; Nurse Cross, 1s.; Nurse McEwan, 5s.; Mrs. Pyne, 5s.; Dr. Bramley, 5s.; total, £7 10s. 6d.

### Wants and Workers.

Miss M. E. Maingay, Havelet, Florence Road, Boscombe, ventures to plead again on behalf of the invalid nurse, as her first appeal met with such a generous response She requires the services of a woman a few hours daily, but the expense is a serious one, as her means are limited, and Miss M. E. Maingay is trying to collect the weekly sum needed. If 36 friends would guarantee 1d. per week, this would make up the required amount.

Will any nurse give her old cotton dresses to a nurse who is shortly going to South Africa to devote her life to the Lepers? Address N. Forrest, 4, Bellevue Parade, Scarborough.

### For Reading to the Sick.

LOVE is born of God, and cannot rest but in God, above all created things. He that loveth . . . giveth all for all, and hath all in all.—*Thos. à Kempis.*

It is not love received
That maketh man to know the inner life
Of them that love him. His own love bestowed
Shall do it! Love thy Father, and no more
His doings shall be strange! —*J. Ingelow.*

Lord, make my heart a place where angels sing!
For surely thoughts low-breathed by Thee
Are angels gliding near on noiseless wing;
And where a home they see
Swept clean, and garnish'd with adoring joy,
They enter in and dwell, and teach that heart to swell
With heavenly melody, their own untired employ.
—*Keble.*

What a strength and spring of life, what hope and trust, what glad, unresting energy, is in this one thought—to serve Him who is "my Lord," ever near me, ever looking on; seeing my intentions before He beholds my failures; knowing my desires before He sees my faults; cheering me to endeavour greater things, and yet accepting the least; inviting my poor service, and yet, above all, content with my poorer love. Let us try to realise this, whatsoever, wheresoever we be. The humblest and the simplest, the weakest and the most encumbered, may love Him not less than the busiest and strongest, the most gifted and laborious. If our heart be clear before Him; if He be to us our chief and sovereign choice, dear above all, and beyond all desired; then all else matters little. That which concerneth us He will perfect in stillness and in power.—*H. E. Manning.*

I would not fain be one
Who, satisfying thirst and breaking fast,
Upon the fulness of the heart, at last
Says no grace after meat.—My wine has run
Indeed out of my cup, and there is none
To gather up the bread of my repast
Scattered and trampled:—yet I find some good
In earth's green herbs and streams that bubble up
Clear from the darkling ground,—content until
I sit with angels before better food.
—*E. B. Browning.*

Offer up to God all pure affections, desires, regrets, and all the bonds which link us to home, kindred, and friends, together with all our works, purposes, and labours. These things, which are not only lawful, but sacred, become then the matter of thanksgiving and oblation. Memories, plans for the future, wishes, intentions; works just begun, half done, all but completed; emotions, sympathies, affections—all these things throng tumultuously and dangerously in the heart and will. The only way to master them is to offer them up to Him, as once ours, under Him, always His by right.—*H. E. Manning.*

### To Nurses.

WE invite contributions from any of our readers, and shall be glad to pay for "Notes on News from the Nursing World," or for articles describing nursing experiences, or dealing with any nursing question from an original point of view. The minimum payment for contributions is 5s., but we welcome interesting contributions of a column, or a page, in length. It may be added that notices of entertainments, presentations, and deaths are not paid for, but, of course, we are always glad to receive them. All rejected manuscripts are returned in due course, and all payments for manuscripts used are made as early as possible at the beginning of each quarter.

### Notes and Queries.

**THE Editor is always willing to answer in this column, without any fee, all reasonable questions, as soon as possible.**

**But the following rules must be carefully observed:—**

**1. Every communication must be accompanied by the name and address of the writer.**

**2. The question must always bear upon nursing, directly or indirectly.**

**If an answer is required by letter a fee of half-a-crown must be enclosed with the note containing the inquiry.**

*Inebriates.*

(141) Would you kindly tell me some particulars of a home for the treatment of inebriates near Liverpool?—*A Nurse.*

Perhaps the Vergmont Sanitorium, 2, Mill Bank, West Derby, Liverpool, would suit you. Apply to the secretary.

*Short Training.*

(142) Would you kindly inform me what post I would be able to obtain after having a twelve months' training as paying probationer in the Liverpool Northern Hospital? Also, would the Cancer Hospital, Stanley Grove, Manchester, be a good training school? It does not mention it in "The Nursing Profession; How and Where to Train."—*Ignor.*

Try and get a three-year certificate; only very subordinate posts are open to those who have so short a training. "The Nursing Profession; How and Where to Train" is compiled with a view to giving intending nurses the information they want, and you had better accept its guidance.

*Older Probationers.*

(143) Would you kindly tell me if there is a recognised training hospital where they will receive nurses 33 years of age. I have nursed for two years in a general hospital, but they did not give certificates, and now I find I am beyond the age limit at most hospitals.—*Dee.*

There are a few which receive probationers up to 35 years of age. See "The Nursing Profession; How and Where to Train." The matron of the Victoria Park Hospital might be able to offer you supplementary training, and secure you a three-year certificate, if your present training is sufficiently good. Write to her fully upon the point.

*District Nursing.*

(144) Nurse L. is a charge nurse, and is anxious to get information about district nursing, pay, hours on duty, and if she boards out.

The standard of district nursing is set by Queen Victoria's Jubilee Institute for Nurses, 1, St. Katherine's Hospital, Regent's Park, N.W., but as all associations frame their regulations according to their individual requirements the rules vary considerably. Apply for information concerning the Q.V.J.I.N. to the General Superintendent.

*Monthly Nursing.*

(145) Some cases have come to my notice lately where the monthly nurse is practically on duty 24 hours in the day. Is the nurse, being properly qualified and L.O.S., ever supposed to have anything to do with the washing of napkins? I shall be much indebted to anyone who will give the best established rules for monthly nurses. I do not like to see any nurse imposed on.—*Nita.*

A monthly nurse's duties are regulated by the exigencies of the case, for it is impossible to lay down strict rules with regard to a procedure which varies so greatly. 2. With regard to washing and needlework &c., these are matters upon which a definite understanding ought to be arrived at before the case is accepted.

*"Queen's Nurses."*

(146) I have had twelve months' training in a private medical home, and am 28 years of age. I should be glad if you would kindly let me know how I can become a "Queen's Nurse."—*Sheffield.*

Apply to the Superintendent of the Queen Victoria Jubilee Institute for Nurses, St. Katherine' Precincts, Gloucester Gate, Regent's Park, London.

(147) Can you kindly tell me what steps are taken to ascertain the condition of those A.N.R. nurses who, in nursing the sick and wounded soldiers in South Africa, have unfortunately been laid low by illness? They are not mentioned in the lists published by the War Office.—*Lady Superintendent, Exeter.*

The War Office authorities inform us that the relatives of nurses, if their addresses are known, are at once communicated with, and there is a special office in South Africa dealing with casualties, in case relatives wish to telegraph or wire direct. We would strongly advise every nurse to constantly carry about with her a paper (such as all soldiers carry) giving her name and address, and the name and address of the relative who is to be informed in case anything happens to her.

*Return from Service.*

(148) Referring to a question in the issue of June 16th, Nurse Sara wishes to thank "A Hospital Reader" for her kind card, and regrets to say it came too late.

**Standard Books of Reference.**

"The Nursing Profession: How and Where to Train." 2s. net.
"The Nurses' Dictionary of Medical Terms." 2s.
"Burdett's Series of Nursing Text-Books." 1s. each.
"A Handbook for Nurses." (Illustrated.) 5s.
"Nursing: Its Theory and Practice." New Edition. 3s. 6d.
"Helps in Sickness and to Health." Fifteenth Thousand. 5s.

All these are published by THE SCIENTIFIC PRESS, Ltd., and may be obtained through any bookseller or direct from the publishers, 28 & 29, Southampton Street, London, W.C.

## Figures and Tables

**Fig. 17. f1:**
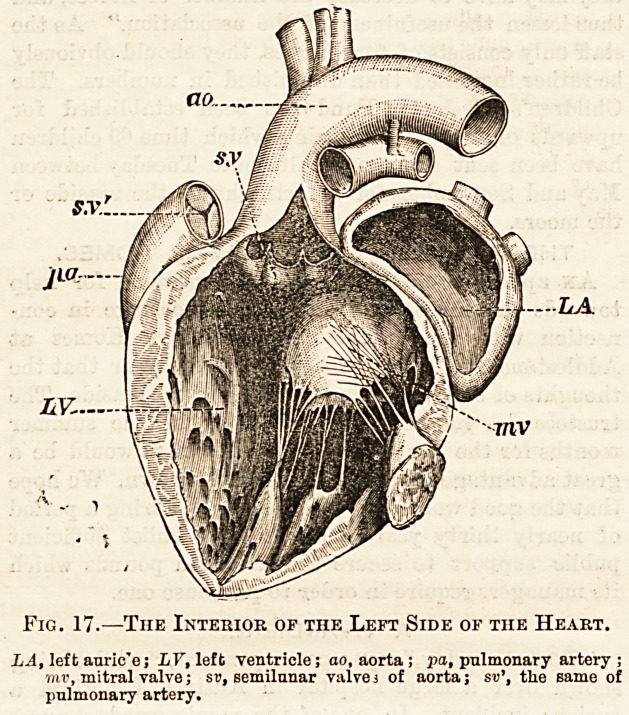


**Fig. 18. f2:**